# 5-(2-Chloro­benz­yl)-4,5,6,7-tetra­hydro­thieno[3,2-*c*]pyridin-2-yl acetate

**DOI:** 10.1107/S1600536812010045

**Published:** 2012-03-14

**Authors:** Jing Yang, Na Chen, Hao Sun, Xiao-Xia Cao, Deng-Ke Liu

**Affiliations:** a Department of Bioengineering, Tianjin Bohai Vocational, and Technical College, Tianjin 300408, People’s Republic of China; b Tianjin Institute of Pharmaceutical Research, Tianjin 300193, People’s Republic of China

## Abstract

In the title compound, C_16_H_16_ClNO_2_S, the benzene and thio­phene rings make a dihedral angle of 72.60 (4)°. In the crystal, weak C—H⋯O inter­actions are observed.

## Related literature

The title compound is a derivative of the anti­platelet agent clopidogrel [systematic name (+)-(*S*)-methyl 2-(2-chloro­phen­yl)-2-(6,7-dihydro­thieno[3,2-*c*]pyridin-5(4*H*)-yl)acetate]. For background to the bioactivity and applications of clopidogrel, see: Muller *et al.* (2003 ▶); Savi *et al.* (1994 ▶); Sharis *et al.* (1998 ▶). For the synthesis of the title compound, see: Roquettes *et al.* (1993 ▶).
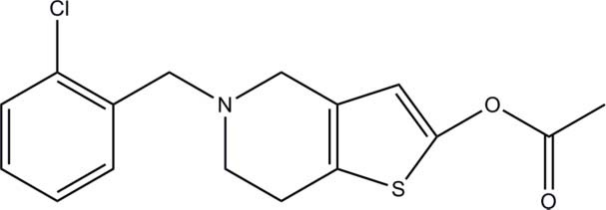


## Experimental

### 

#### Crystal data

C_16_H_16_ClNO_2_S
*M* *_r_* = 321.81
Monoclinic, 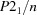

*a* = 14.526 (3) Å
*b* = 6.1065 (12) Å
*c* = 17.490 (3) Å
β = 99.098 (3)°
*V* = 1532.0 (5) Å^3^
*Z* = 4
Mo *K*α radiation
μ = 0.39 mm^−1^
*T* = 113 K
0.20 × 0.18 × 0.10 mm

#### Data collection

Rigaku Saturn CCD area-detector diffractometer
Absorption correction: multi-scan (*CrystalClear*; Rigaku/MSC, 2005 ▶) *T*_min_ = 0.926, *T*_max_ = 0.962
10686 measured reflections
2704 independent reflections
2397 reflections with *I* > 2σ(*I*)
*R*_int_ = 0.033

#### Refinement

*R*[*F* ^2^ > 2σ(*F* ^2^)] = 0.030
*wR*(*F* ^2^) = 0.086
*S* = 1.07
2704 reflections
191 parameters
H-atom parameters constrained
Δρ_max_ = 0.18 e Å^−3^
Δρ_min_ = −0.31 e Å^−3^

### 

Data collection: *CrystalClear* (Rigaku/MSC, 2005 ▶); cell refinement: *CrystalClear*; data reduction: *CrystalClear*; program(s) used to solve structure: *SHELXS97* (Sheldrick, 2008 ▶); program(s) used to refine structure: *SHELXL97* (Sheldrick, 2008 ▶); molecular graphics: *SHELXTL* (Sheldrick, 2008 ▶); software used to prepare material for publication: *CrystalStructure* (Rigaku/MSC, 2005 ▶).

## Supplementary Material

Crystal structure: contains datablock(s) I, global. DOI: 10.1107/S1600536812010045/kp2393sup1.cif

Structure factors: contains datablock(s) I. DOI: 10.1107/S1600536812010045/kp2393Isup2.hkl

Supplementary material file. DOI: 10.1107/S1600536812010045/kp2393Isup3.cdx

Supplementary material file. DOI: 10.1107/S1600536812010045/kp2393Isup4.cml

Additional supplementary materials:  crystallographic information; 3D view; checkCIF report

## Figures and Tables

**Table 1:** Hydrogen-bond geometry (Å, °)

*D*—H⋯*A*	*D*—H	H⋯*A*	*D*⋯*A*	*D*—H⋯*A*
C15—H15⋯O1^i^	0.95	2.52	3.364 (2)	148

Symmetry code: (i) 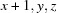.

**Fig. 1.**
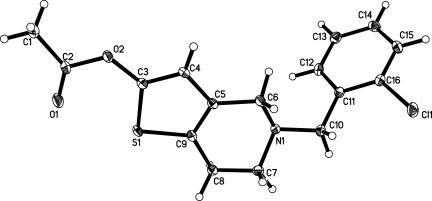
The molecular structure of (I), with the atom-numbering scheme and 50% probability displacement ellipsoids.

## supplementary crystallographic information

### Comment

Clopidogrel is an oral, thienopyridine class of antiplatelet agent used to inhibit blood clots in coronary artery disease, peripheral vascular disease, and cerebrovascular disease (Muller *et al.*, 2003; Savi *et al.*, 1994; Sharis *et al.*, 1998). The molecular structure of the title compound, a derivative of clopidogrel, is reported here. The thiophene and benzene rings make a dihedral angle of 72.60 (4)°; the tetrahydropyridine ring adopts a half-chair conformation (Fig. 1). In the crystal structure, the packing is realsied by weak intramolecular C—H···Cl and C—H···N, and intermolecular C—H···O interaction (Table 1).

### Experimental

We used the method of Roquettes *et al.* (1993) to sythesize the title compound. 8.85 g (0.0316 mol) of 5-(2-chlorobenzyl)-5,6,7,7a-tetrahydro-4*H*-thieno [3,2-*c*] pyridine-2-one are dissolved in 120 mL of isopropenyl acetate with 7.8 g (0.0411 mol) of *p*-toluenesulphonic acid; the medium is stirred at 363 K for 6 h. After cooling to about 293 K, 2 volumes of water are introduced into the medium, the pH is made basic by adding saturated aqueous NaHCO_3_ solution and the desired product is extracted with ethyl acetate. After removal of the solvent, the oil, dissolved in CH_2_Cl_2_, is filtered on silica to give a 68% yield of the target compound. Colourless single crystals were grown from a methanol solution.

### Refinement

All H atoms were positioned geometrically and refined using a riding model, with d(C—H) = 0.95 - 0.99 Å, and *U*_iso_(H) = 1.5 or 1.2*U*_eq_.

### Crystal data

**Table:**

C_16_H_16_ClNO_2_S	*F*(000) = 672
*M**_r_* = 321.81	*D*_x_ = 1.395 Mg m^−^^3^
Monoclinic, *P*2_1_/*n*	Mo *K*α radiation, λ = 0.71073 Å
Hall symbol: -P 2yn	Cell parameters from 4955 reflections
*a* = 14.526 (3) Å	θ = 1.7–27.9°
*b* = 6.1065 (12) Å	µ = 0.39 mm^−^^1^
*c* = 17.490 (3) Å	*T* = 113 K
β = 99.098 (3)°	Prism, colourless
*V* = 1532.0 (5) Å^3^	0.20 × 0.18 × 0.10 mm
*Z* = 4	

### Data collection

**Table:**

Rigaku Saturn CCD area-detector diffractometer	2704 independent reflections
Radiation source: rotating anode	2397 reflections with *I* > 2σ(*I*)
Multilayer monochromator	*R*_int_ = 0.033
Detector resolution: 14.63 pixels mm^-1^	θ_max_ = 25.0°, θ_min_ = 1.7°
ω and φ scans	*h* = −17→17
Absorption correction: multi-scan (*CrystalClear*; Rigaku/MSC, 2005)	*k* = −7→5
*T*_min_ = 0.926, *T*_max_ = 0.962	*l* = −20→20
10686 measured reflections	

### Refinement

**Table:**

Refinement on *F*^2^	Primary atom site location: structure-invariant direct methods
Least-squares matrix: full	Secondary atom site location: difference Fourier map
*R*[*F*^2^ > 2σ(*F*^2^)] = 0.030	Hydrogen site location: inferred from neighbouring sites
*wR*(*F*^2^) = 0.086	H-atom parameters constrained
*S* = 1.07	*w* = 1/[σ^2^(*F*_o_^2^) + (0.0514*P*)^2^ + 0.2496*P*] where *P* = (*F*_o_^2^ + 2*F*_c_^2^)/3
2704 reflections	(Δ/σ)_max_ = 0.001
191 parameters	Δρ_max_ = 0.18 e Å^−^^3^
0 restraints	Δρ_min_ = −0.31 e Å^−^^3^

### Special details

**Table:**

Geometry. All e.s.d.'s (except the e.s.d. in the dihedral angle between two l.s. planes) are estimated using the full covariance matrix. The cell e.s.d.'s are taken into account individually in the estimation of e.s.d.'s in distances, angles and torsion angles; correlations between e.s.d.'s in cell parameters are only used when they are defined by crystal symmetry. An approximate (isotropic) treatment of cell e.s.d.'s is used for estimating e.s.d.'s involving l.s. planes.
Refinement. Refinement of *F*^2^ against ALL reflections. The weighted *R*-factor *wR* and goodness of fit *S* are based on *F*^2^, conventional *R*-factors *R* are based on *F*, with *F* set to zero for negative *F*^2^. The threshold expression of *F*^2^ > σ(*F*^2^) is used only for calculating *R*-factors(gt) *etc*. and is not relevant to the choice of reflections for refinement. *R*-factors based on *F*^2^ are statistically about twice as large as those based on *F*, and *R*- factors based on ALL data will be even larger.

### Fractional atomic coordinates and isotropic or equivalent isotropic displacement parameters (Å^2^)

**Table:**

	*x*	*y*	*z*	*U*_iso_*/*U*_eq_	
Cl1	0.64701 (3)	0.34141 (7)	0.05214 (2)	0.03277 (14)	
S1	0.08191 (2)	0.16665 (6)	0.14645 (2)	0.01970 (13)	
O1	−0.07808 (8)	−0.0621 (2)	0.12690 (7)	0.0350 (3)	
O2	0.03009 (7)	−0.21624 (17)	0.06530 (6)	0.0227 (2)	
N1	0.38577 (8)	0.2952 (2)	0.15330 (6)	0.0197 (3)	
C1	−0.11190 (11)	−0.4038 (3)	0.05983 (9)	0.0262 (4)	
H1A	−0.1710	−0.4020	0.0802	0.039*	
H1B	−0.0773	−0.5373	0.0771	0.039*	
H1C	−0.1245	−0.4005	0.0031	0.039*	
C2	−0.05566 (10)	−0.2093 (3)	0.08876 (8)	0.0232 (3)	
C3	0.09637 (10)	−0.0575 (2)	0.08845 (8)	0.0189 (3)	
C4	0.18265 (10)	−0.0674 (2)	0.06854 (8)	0.0192 (3)	
H4	0.2022	−0.1776	0.0362	0.023*	
C5	0.24081 (10)	0.1077 (2)	0.10193 (7)	0.0180 (3)	
C6	0.34116 (10)	0.1424 (2)	0.09400 (8)	0.0209 (3)	
H6A	0.3745	0.0004	0.0994	0.025*	
H6B	0.3451	0.2017	0.0419	0.025*	
C7	0.32906 (10)	0.4937 (3)	0.15410 (8)	0.0212 (3)	
H7A	0.3132	0.5520	0.1008	0.025*	
H7B	0.3651	0.6070	0.1865	0.025*	
C8	0.23974 (10)	0.4424 (2)	0.18630 (8)	0.0205 (3)	
H8A	0.2543	0.4127	0.2426	0.025*	
H8B	0.1965	0.5686	0.1782	0.025*	
C9	0.19592 (10)	0.2454 (3)	0.14461 (8)	0.0187 (3)	
C10	0.47954 (10)	0.3512 (2)	0.13892 (9)	0.0243 (4)	
H10A	0.5017	0.4817	0.1701	0.029*	
H10B	0.4764	0.3902	0.0836	0.029*	
C11	0.54919 (10)	0.1683 (2)	0.15836 (8)	0.0199 (3)	
C12	0.54030 (10)	0.0141 (3)	0.21553 (8)	0.0245 (3)	
H12	0.4874	0.0207	0.2412	0.029*	
C13	0.60608 (11)	−0.1481 (3)	0.23609 (9)	0.0258 (4)	
H13	0.5982	−0.2505	0.2754	0.031*	
C14	0.68378 (11)	−0.1607 (3)	0.19890 (9)	0.0264 (4)	
H14	0.7289	−0.2724	0.2125	0.032*	
C15	0.69503 (11)	−0.0102 (3)	0.14218 (8)	0.0259 (4)	
H15	0.7480	−0.0172	0.1166	0.031*	
C16	0.62835 (10)	0.1509 (2)	0.12296 (8)	0.0208 (3)	

### Atomic displacement parameters (Å^2^)

**Table:**

	*U*^11^	*U*^22^	*U*^33^	*U*^12^	*U*^13^	*U*^23^
Cl1	0.0313 (2)	0.0369 (3)	0.0341 (2)	0.00271 (18)	0.01724 (18)	0.01129 (18)
S1	0.0156 (2)	0.0234 (2)	0.0206 (2)	0.00371 (15)	0.00426 (15)	−0.00402 (15)
O1	0.0202 (6)	0.0410 (7)	0.0459 (7)	−0.0015 (5)	0.0117 (5)	−0.0193 (6)
O2	0.0158 (5)	0.0253 (6)	0.0273 (5)	0.0010 (4)	0.0048 (4)	−0.0066 (5)
N1	0.0158 (6)	0.0185 (6)	0.0250 (7)	0.0012 (5)	0.0039 (5)	0.0012 (5)
C1	0.0193 (8)	0.0292 (9)	0.0295 (8)	−0.0003 (7)	0.0019 (6)	−0.0017 (7)
C2	0.0144 (7)	0.0307 (9)	0.0240 (7)	0.0029 (7)	0.0017 (6)	0.0001 (7)
C3	0.0178 (7)	0.0210 (8)	0.0176 (7)	0.0025 (6)	0.0014 (5)	−0.0023 (6)
C4	0.0187 (8)	0.0201 (8)	0.0189 (7)	0.0054 (6)	0.0031 (6)	−0.0010 (6)
C5	0.0177 (7)	0.0200 (7)	0.0161 (7)	0.0043 (6)	0.0021 (5)	0.0027 (6)
C6	0.0197 (8)	0.0215 (8)	0.0224 (7)	0.0032 (6)	0.0061 (6)	0.0005 (6)
C7	0.0229 (8)	0.0185 (8)	0.0223 (7)	0.0012 (6)	0.0042 (6)	0.0007 (6)
C8	0.0206 (8)	0.0211 (8)	0.0206 (7)	0.0024 (6)	0.0057 (6)	−0.0016 (6)
C9	0.0155 (7)	0.0226 (8)	0.0178 (7)	0.0027 (6)	0.0028 (5)	0.0015 (6)
C10	0.0180 (8)	0.0233 (8)	0.0325 (8)	−0.0011 (6)	0.0063 (6)	0.0047 (7)
C11	0.0166 (7)	0.0210 (8)	0.0212 (7)	−0.0030 (6)	0.0003 (6)	−0.0018 (6)
C12	0.0206 (8)	0.0282 (9)	0.0256 (7)	−0.0031 (7)	0.0069 (6)	0.0022 (7)
C13	0.0265 (9)	0.0251 (9)	0.0251 (8)	−0.0022 (7)	0.0019 (6)	0.0048 (7)
C14	0.0219 (8)	0.0252 (9)	0.0302 (8)	0.0046 (7)	−0.0015 (6)	−0.0015 (7)
C15	0.0199 (8)	0.0315 (9)	0.0268 (8)	0.0018 (7)	0.0047 (6)	−0.0036 (7)
C16	0.0212 (8)	0.0227 (8)	0.0187 (7)	−0.0045 (6)	0.0038 (6)	−0.0003 (6)

### Geometric parameters (Å, º)

**Table:**

Cl1—C16	1.7515 (15)	C7—C8	1.526 (2)
S1—C9	1.7298 (15)	C7—H7A	0.9900
S1—C3	1.7362 (14)	C7—H7B	0.9900
O1—C2	1.1946 (19)	C8—C9	1.496 (2)
O2—C2	1.3722 (17)	C8—H8A	0.9900
O2—C3	1.3810 (18)	C8—H8B	0.9900
N1—C10	1.4641 (18)	C10—C11	1.509 (2)
N1—C7	1.4671 (19)	C10—H10A	0.9900
N1—C6	1.4676 (19)	C10—H10B	0.9900
C1—C2	1.485 (2)	C11—C16	1.393 (2)
C1—H1A	0.9800	C11—C12	1.394 (2)
C1—H1B	0.9800	C12—C13	1.384 (2)
C1—H1C	0.9800	C12—H12	0.9500
C3—C4	1.354 (2)	C13—C14	1.391 (2)
C4—C5	1.429 (2)	C13—H13	0.9500
C4—H4	0.9500	C14—C15	1.381 (2)
C5—C9	1.358 (2)	C14—H14	0.9500
C5—C6	1.501 (2)	C15—C16	1.384 (2)
C6—H6A	0.9900	C15—H15	0.9500
C6—H6B	0.9900		
			
C9—S1—C3	90.21 (7)	H7A—C7—H7B	108.1
C2—O2—C3	120.95 (12)	C9—C8—C7	107.84 (12)
C10—N1—C7	110.35 (12)	C9—C8—H8A	110.1
C10—N1—C6	110.24 (11)	C7—C8—H8A	110.1
C7—N1—C6	110.26 (11)	C9—C8—H8B	110.1
C2—C1—H1A	109.5	C7—C8—H8B	110.1
C2—C1—H1B	109.5	H8A—C8—H8B	108.5
H1A—C1—H1B	109.5	C5—C9—C8	124.19 (13)
C2—C1—H1C	109.5	C5—C9—S1	112.48 (11)
H1A—C1—H1C	109.5	C8—C9—S1	123.33 (11)
H1B—C1—H1C	109.5	N1—C10—C11	113.41 (12)
O1—C2—O2	122.06 (14)	N1—C10—H10A	108.9
O1—C2—C1	127.46 (14)	C11—C10—H10A	108.9
O2—C2—C1	110.48 (13)	N1—C10—H10B	108.9
C4—C3—O2	121.60 (13)	C11—C10—H10B	108.9
C4—C3—S1	112.82 (11)	H10A—C10—H10B	107.7
O2—C3—S1	125.56 (10)	C16—C11—C12	116.34 (14)
C3—C4—C5	111.90 (13)	C16—C11—C10	121.82 (13)
C3—C4—H4	124.1	C12—C11—C10	121.76 (13)
C5—C4—H4	124.1	C13—C12—C11	122.09 (14)
C9—C5—C4	112.57 (13)	C13—C12—H12	119.0
C9—C5—C6	121.40 (13)	C11—C12—H12	119.0
C4—C5—C6	126.01 (13)	C12—C13—C14	119.74 (14)
N1—C6—C5	110.56 (11)	C12—C13—H13	120.1
N1—C6—H6A	109.5	C14—C13—H13	120.1
C5—C6—H6A	109.5	C15—C14—C13	119.75 (15)
N1—C6—H6B	109.5	C15—C14—H14	120.1
C5—C6—H6B	109.5	C13—C14—H14	120.1
H6A—C6—H6B	108.1	C14—C15—C16	119.31 (14)
N1—C7—C8	110.19 (12)	C14—C15—H15	120.3
N1—C7—H7A	109.6	C16—C15—H15	120.3
C8—C7—H7A	109.6	C15—C16—C11	122.76 (14)
N1—C7—H7B	109.6	C15—C16—Cl1	117.60 (12)
C8—C7—H7B	109.6	C11—C16—Cl1	119.63 (12)
			
C3—O2—C2—O1	3.4 (2)	C6—C5—C9—S1	−178.61 (10)
C3—O2—C2—C1	−176.85 (12)	C7—C8—C9—C5	15.76 (19)
C2—O2—C3—C4	177.09 (13)	C7—C8—C9—S1	−164.89 (10)
C2—O2—C3—S1	−1.09 (19)	C3—S1—C9—C5	0.20 (11)
C9—S1—C3—C4	−0.86 (11)	C3—S1—C9—C8	−179.22 (12)
C9—S1—C3—O2	177.46 (12)	C7—N1—C10—C11	164.39 (12)
O2—C3—C4—C5	−177.12 (12)	C6—N1—C10—C11	−73.58 (15)
S1—C3—C4—C5	1.27 (15)	N1—C10—C11—C16	154.60 (13)
C3—C4—C5—C9	−1.12 (17)	N1—C10—C11—C12	−28.7 (2)
C3—C4—C5—C6	177.91 (13)	C16—C11—C12—C13	−0.2 (2)
C10—N1—C6—C5	−172.40 (12)	C10—C11—C12—C13	−177.04 (14)
C7—N1—C6—C5	−50.32 (15)	C11—C12—C13—C14	−0.3 (2)
C9—C5—C6—N1	15.88 (18)	C12—C13—C14—C15	0.5 (2)
C4—C5—C6—N1	−163.07 (12)	C13—C14—C15—C16	−0.2 (2)
C10—N1—C7—C8	−167.59 (12)	C14—C15—C16—C11	−0.2 (2)
C6—N1—C7—C8	70.39 (14)	C14—C15—C16—Cl1	178.66 (11)
N1—C7—C8—C9	−49.62 (14)	C12—C11—C16—C15	0.5 (2)
C4—C5—C9—C8	179.88 (12)	C10—C11—C16—C15	177.29 (14)
C6—C5—C9—C8	0.8 (2)	C12—C11—C16—Cl1	−178.42 (11)
C4—C5—C9—S1	0.47 (16)	C10—C11—C16—Cl1	−1.6 (2)

### Hydrogen-bond geometry (Å, º)

**Table:**

*D*—H···*A*	*D*—H	H···*A*	*D*···*A*	*D*—H···*A*
C10—H10*B*···Cl1	0.99	2.64	3.0648 (16)	106
C12—H12···N1	0.95	2.58	2.898 (2)	100
C15—H15···O1^i^	0.95	2.52	3.364 (2)	148

Symmetry code: (i) *x*+1, *y*, *z*.

## References

[bb2] Muller, I., Besta, F., Schulz, C., Li, Z., Massberg, S. & Gawaz, M. (2003). *Circulation*, **108**, 2195–2197. 10.1161/01.CIR.0000099507.32936.C0 14568892

[bb3] Rigaku/MSC (2005). *CrystalClear* and *CrystalStructure* Rigaku/MSC Inc., The Woodlands, Texas, USA.

[bb1] Roquettes, B. A., Bordes, M. F., sur Seze, L., Toulouse, D. F., Herbert, J. M. & du Touch, P. (1993). US Patent No. 5 190 938.

[bb4] Savi, P., Combalbert, J., Gaich, C., Rouchon, M. C., Maffrand, J. P., Berger, Y. & Herbert, J. M. (1994). *Thromb. Haemost.* **72**, 313–317. 7831671

[bb5] Sharis, P. J., Cannon, C. P. & Loscalzo, J. (1998). *Ann. Intern. Med.* **129**, 394–405. 10.7326/0003-4819-129-5-199809010-00009 9735068

[bb6] Sheldrick, G. M. (2008). *Acta Cryst.* A**64**, 112–122. 10.1107/S0108767307043930 18156677

